# Sirtuin 3 mutation- induced mitochondrial dysfunction and optic neuropathy: a case report

**DOI:** 10.1186/s12886-023-02872-x

**Published:** 2023-03-24

**Authors:** Bo Young Chun, Jung Moon Choi, Su-Kyeong Hwang, Soolienah Rhiu

**Affiliations:** 1 Department of Ophthalmology, School of Medicine, Kyungpook National University, Daegu, Korea; 2 Brain Science & Engineering Institute, School of Medicine, Kyungpook National University, 680 Gukchaebosang Street, 700-422 Daegu, South Korea; 3 Department of Pediatrics, School of Medicine, Kyungpook National University, Daegu, South Korea; 4 Department of Ophthalmology, Dongtan Sacred Heart Hospital, Hallym University College of Medicine, Hwaswong, Korea

**Keywords:** Mitochondrial dysfunction, Mitochondrial optic neuropathy, *SIRT3* gene mutation, Case report

## Abstract

**Background:**

Mitochondrial optic neuropathy is characterized by painless, progressive, symmetrical central vision loss, and dyschromatopsia owing to mitochondrial dysfunction. This report documents a rare case of mitochondrial optic neuropathy due to the *SIRT3* gene mutation.

**Case presentation:**

This report describes a case of a 17-year-old boy who presented with symptoms of bilateral painless, progressive vision decline over several years. Fundus examination revealed temporal pallor of the optic nerve head in both the eyes and an OCT showed considerable thinning of the retinal nerve fiber and ganglion cell layers. Pathogenicity was confirmed by decreased mitochondrial function measured by bioenergetic health index and oxygen consumption rate in this patient. Subsequent NGS revealed a missense mutation of the *SIRT3* gene (c.1137G > C, p.Trp379Cys) in the patient.

**Conclusions:**

This case describes the clinical manifestation of mitochondrial optic neuropathy due to the *SIRT3* gene mutation.

## Background

Mitochondria produce energy through oxidative phosphorylation and are indispensable in the life of cells, especially neurons [1]. Since the visual system’s function relies mainly on the mitochondrial energy supply, the eye is one of the organs with the densest mitochondrial population in the body [1]. Retinal ganglion cells (RGCs) and their axons are preferentially vulnerable to neurodegeneration due to mitochondrial dysfunction [1]. Mitochondrial optic neuropathy is characterized by painless, progressive, symmetrical central vision loss, and dyschromatopsia owing to mitochondrial dysfunction. Leber’s hereditary optic neuropathy and dominant optic atrophy (DOA) are the two most common inherited optic neuropathies related to mitochondrial dysfunction observed in clinical practice [2]. However, mutations of any genes, which affect mitochondrial function, can theoretically induce mitochondrial optic neuropathy. Here, we present a rare case of mitochondrial optic neuropathy from a missense mutation of the sirtuin 3 (*SIRT3*) gene.

## Case presentation

**Fig. 1:**
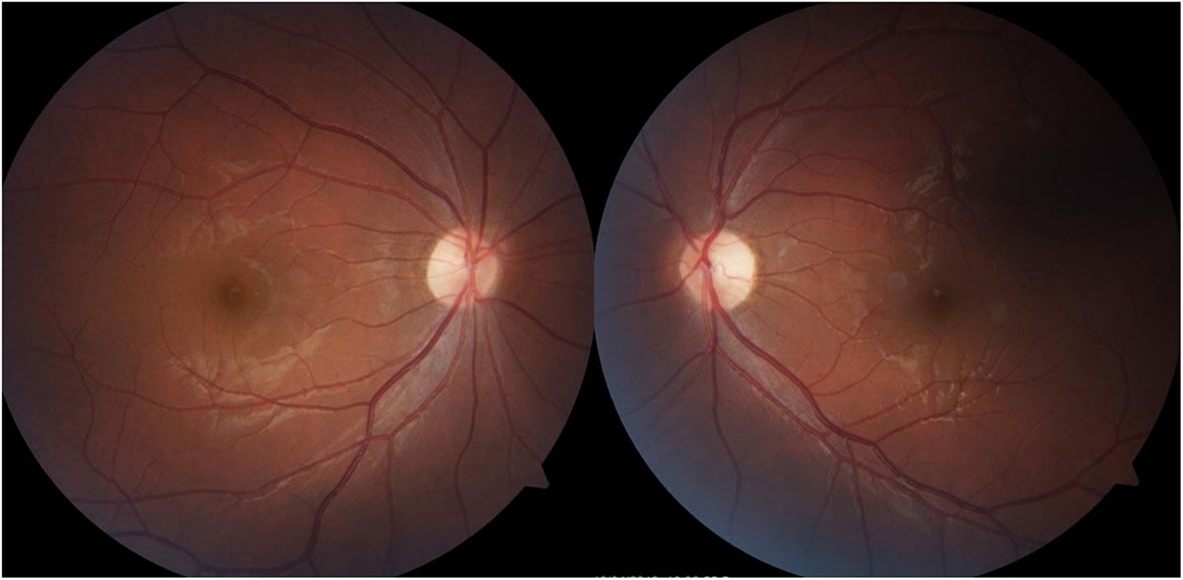
Fundus photography of the patient. Fundus examination showed temporal pallor of the optic nerve head in both eyes

**Fig. 2:**
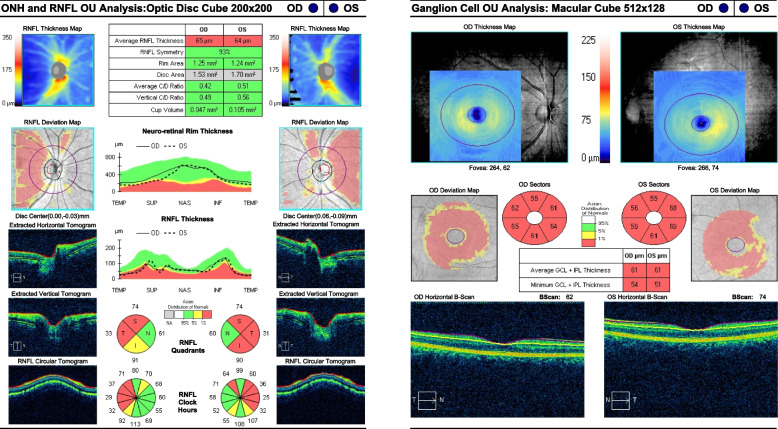
Optical coherence tomography of the patient. It showed considerable thinning of the peripapillary retinal nerve fiber layer and macular ganglion cell layers, which demonstrated preferential loss of the the papillomacular bundle in both eyes

**Fig. 3:**
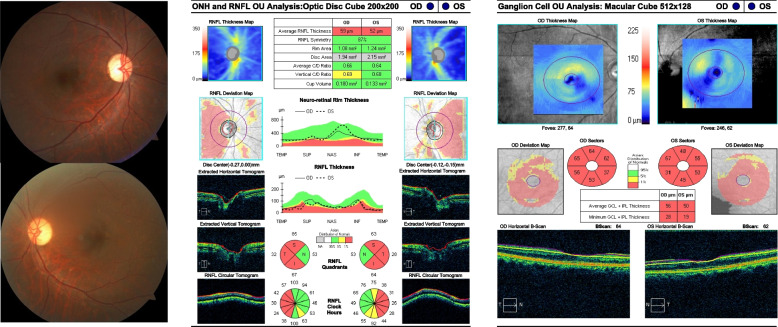
Fundus photography and Optical coherence tomogtaphy of the patient’s mother. Fundus examination showed temporal pallor of the optic nerve head in both eyes. Optical coherence tomography showed considerable thinning of the peripapillary retinal nerve fiber layer and macular ganglion cell layers in both eyes

A 17-year-old boy presented with a primary complaint of bilateral painless, progressive vision decline over several years. Except for dyschromatopsia, his past medical, ocular, and neurological symptoms were unremarkable. There was no history of trauma or medication intake, and he did not smoke or drink. His father and sister were healthy and had no vision-related problems. However, his mother seemed to have mild mental retardation. The best-corrected visual acuity (BCVA) in both eyes of the patients was 20/80, without any relative afferent pupillary defect. He was orthophoric and demonstrated full duction and version. He identified three Ishihara color plates with each eye. The findings of the slit lamp examination, including measurement of the intraocular pressure were normal; however, fundus examination revealed temporal pallor of the optic nerve head in both eyes (Fig. 1). The Goldmann visual field test demonstrated cecocentral scotomas in both the eyes, and optical coherence tomography (OCT) showed considerable thinning of the peripapillary retinal nerve fiber layer (RNFL) and macular ganglion cell layers, which demonstrated preferential loss of the the papillomacular bundle in both eyes (Fig. 2). However, the electrophysiology retinogram revealed normal. Orbital magnetic resonance imaging with gadolinium enhancement demonstrated no abnormal findings. Genetic testing by whole genome sequencing of the mitochondrial DNA was normal. However, next-generation sequencing of the optic atrophy panel revealed a missense mutation of the *SIRT3* gene (c.1137G > C, p.Trp379Cys) in the patient and his mother. BCVA of his mother was 20/32 in both eyes without any relative afferent pupillary defect. She identified six Ishhara color plate with each eye. Her fundus examination revealed temporal pallor of the optic nerve head in both eyes, and OCT revealed thinning of the peripapillary RNFL and macular ganglion cell layers of both eyes (Fig. 3). The Goldmann visual field test was not available due to her poor cooperation.

**Fig. 4:**
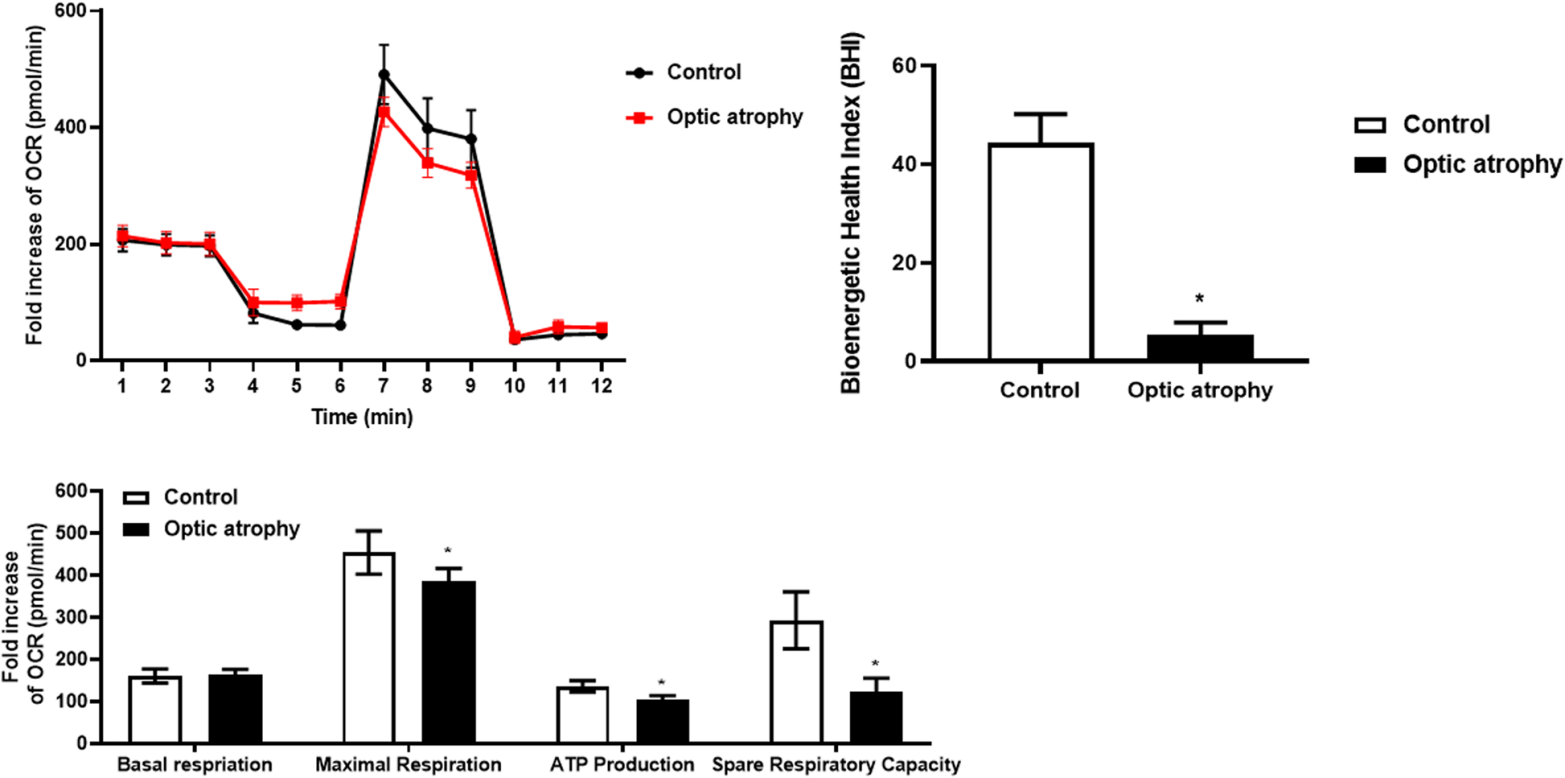
Mitochondrial function measurement in the patient’s peripheral blood mononuclear cells with the Seahorse XF Cell Mito Stress Test kit (Agilent, Santa Clara, USA) revealed a significant decrease in the function measured as a bioenergetic health index (BHI) and oxygen consumption rate (OCR) compared with those of age and sex matched healthy control

Mitochondrial function measurement in the patient’s peripheral blood mononuclear cells with the Seahorse XF Cell Mito Stress Test kit (Agilent, Santa Clara, USA) revealed a significant decrease in the function measured as a bioenergetic health index (BHI) and oxygen consumption rate (OCR) compared with those of age and sex matched healthy control (Fig. 4). Two years after the initial presentation, the BCVA in both eyes decreased to 20/100 and 20/125 each, and he demonstrated temporal optic nerve head pallor and stabilized RNFL thinning on OCT.

## Discussion and conclusions

Sirtuins are a family of seven proteins with nicotinamide adenine dinucleotide-dependent deacetylase activity that regulate multiple cellular processes, such as stress resistance, apoptosis, inflammation, and mitochondrial energy homeostasis [3]. In mammals, *SIRT3*, *4*, and *5* are localized in the mitochondrial matrix and modulate mitochondrial metabolism, but it is the *SIRT3* that functions uniquely as a major regulator of the whole organelle acetylome [3]. It is a key modulator in maintaining mitochondrial integrity and functions by regulating the metabolic enzyme activity and mitochondrial fusion by activation of the optic atrophy 1 (*OPA1*) [3, 4]. *OPA1*, reported as a direct target of *SIRT3*, is hyperacetylated, and its GTPase activity diminishes under stress conditions [3, 4]. *SIRT3* deacetylates and activates *OPA1* and fine-tunes mitochondrial dynamics during cellular stress [3, 4]. *SIRT3* silencing can lead to hyperacetylation of mitochondrial proteins and mitochondrial dysfunction [3, 4]. Therefore, *SIRT3* is recognized as the major mitochondrial deacetylase.

DOA is the most commonly diagnosed hereditary optic atrophy, causing progressive bilateral vision loss which begins early in life [1, 2]. The *OPA1* gene is the most common gene mutated in DOA and accounts for 65–90% of all cases of DOA [1, 2]. Recently, Rocatcher et al. [5] reported the top 10 most frequently involved genes in hereditary optic neuropathies; the *OPA1* gene was the first position in the top 10 nuclear genes, followed by *WFS1, ACO2, SPG7, MFN2, AFG3L2, RTN4IP1, TMEM126A, NR2F1* and *FDXR*. Their results revealed that those 10 genes accounted for 96% of the cases of hereditary optic neuropathies, and the majority of the implicated genes directly linked to mitochondrial function [5]. A study on the application of next-generation sequencing in suspected hereditary optic atrophy in Korean also supports the *OPA1* variants as being the major genetic causes [6]. They reported that the *OPA1* variants were found to be the major causes of hereditary optic atrophy, accounting for 38.9% of their cases [6].

Our patient presented with typical manifestations of DOA, i.e., progressive, symmetrical, painless, bilateral vision loss, and dyschromatopsia. Examinations revealed the temporal pallor of the optic nerve head and selective thinning of the peripapillary RNFL and macular ganglion cell layers. Loss of *OPA1* protein function by *OPA1* gene mutations leads to mitochondrial dysfunction that eventually causes apoptosis of the RGCs in patients with DOA [2]. Therefore, we clinically diagnosed the patient with DOA and hypothesized that he might have mutations in the *OPA1* gene or *OPA1* interacting proteins. Although he presented with typical ophthalmologic manifestations of mitochondrial optic neuropathy, his mother presented with a relatively better vision than his son, despite having identical *SIRT3* mutation. This difference in clinical severity might be explained by sex-specific susceptibility to visual defects due to mitochondrial dysfunction [7]. Molecular diagnosis was achieved by next-generation sequencing but suspected based on the clinical phenotype. Pathogenicity was confirmed by decreased mitochondrial function measured by BHI and OCR in this patient. This finding corresponds with a previous study that reported that reduced basal OCR was observed in skeletal muscles of the *SIRT3* knockout mice [4]. Recent findings show that *SIRT3* activity is vital to the expression levels of *OPA1*, and *SIRT3* can directly interact with and deacetylate *OPA1* [3, 4]. *SIRT3*- induced *OPA1* deacetylation can increase its GTPase activity to promote the fusion of the inner mitochondrial membrane and maintain mitochondrial dynamics [3, 4].

Consistent with these reports, our case suggests that *SIRT3* mutation can induce mitochondrial optic neuropathy caused by mitochondrial dysfunction. To the best of our knowledge, this is the first report of mitochondrial optic neuropathy from the missense mutation of the *SIRT3* gene. This case report emphasizes that not only mutations of *OPA1* gene but also *OPA1* interacting proteins may cause mitochondrial optic neuropathy.

## Data Availability

The datasets used during the current study are available from the corresponding authors on reasonable request.

## Abbreviations

BCVA: best-corrected visual acuity
BHI: bioenergetic health index
DOA: dominant optic atrophy
OCR: oxygen consumption rate
OCT: optical coherence tomography
*OPA1*: optic atrophy 1
RGCs: retinal ganglion cells
RNFL: retinal nerve fiber layer
*SIRT3*: *sirtuin 3*
